# Beyond static descriptors: the need for *operando* insights into MXene catalysts for the hydrogen evolution reaction

**DOI:** 10.1039/d6cp02227h

**Published:** 2026-10-15

**Authors:** Jahid Uz Zaman, Johan E. ten Elshof, Christoph Baeumer

**Affiliations:** a MESA+ Institute, Faculty of Science and Technology, University of Twente Enschede Netherlands c.baeumer@utwente.nl

## Abstract

MXenes have emerged as promising electrocatalysts for the hydrogen evolution reaction (HER) owing to their tunable electronic structure, diverse surface terminations, and high electrical conductivity. Over the past decade, numerous theoretical studies have proposed activity descriptors to rationalize HER performance on MXene surfaces, including hydrogen adsorption free energy, surface termination composition, electronic structure of transition-metal centers, and defect formation. While these descriptors provide useful guidelines for understanding catalytic trends, they are typically derived from static surface models or *ex situ* characterization, which may not accurately represent the dynamic catalyst–electrolyte interface under operating electrochemical conditions. During HER, interactions with the electrolyte, applied potential, and reaction intermediates can modify surface terminations, alter metal electronic structure, reorganize interfacial water networks, and generate new defect sites. Such processes can significantly influence catalytic activity and challenge the validity of descriptors defined for idealized surfaces. In this review, we discuss the current understanding of activity descriptors for MXene-based HER electrocatalysts and highlight the importance of probing the active interface under reaction conditions. Particular emphasis is placed on the role of *operando* spectroscopic techniques, including Raman spectroscopy, infrared spectroscopy, X-ray absorption spectroscopy, and photoelectron spectroscopy, in directly monitoring the evolution of surface chemistry and electronic structure during catalysis. By bridging theoretical predictions with experimental observations, *operando* investigations provide critical insight into the dynamic nature of MXene catalytic interfaces and offer pathways for validating descriptor–activity relationships. These advances are expected to guide the rational design of next-generation MXene electrocatalysts for efficient hydrogen production.

## Abstract 2

Understanding HER activity on MXenes requires probing their evolving solid–liquid interface. *Operando* spectroscopy can link dynamic surface chemistry, electronic structure, and interfacial water to catalytic performance.

## Graphical abstract

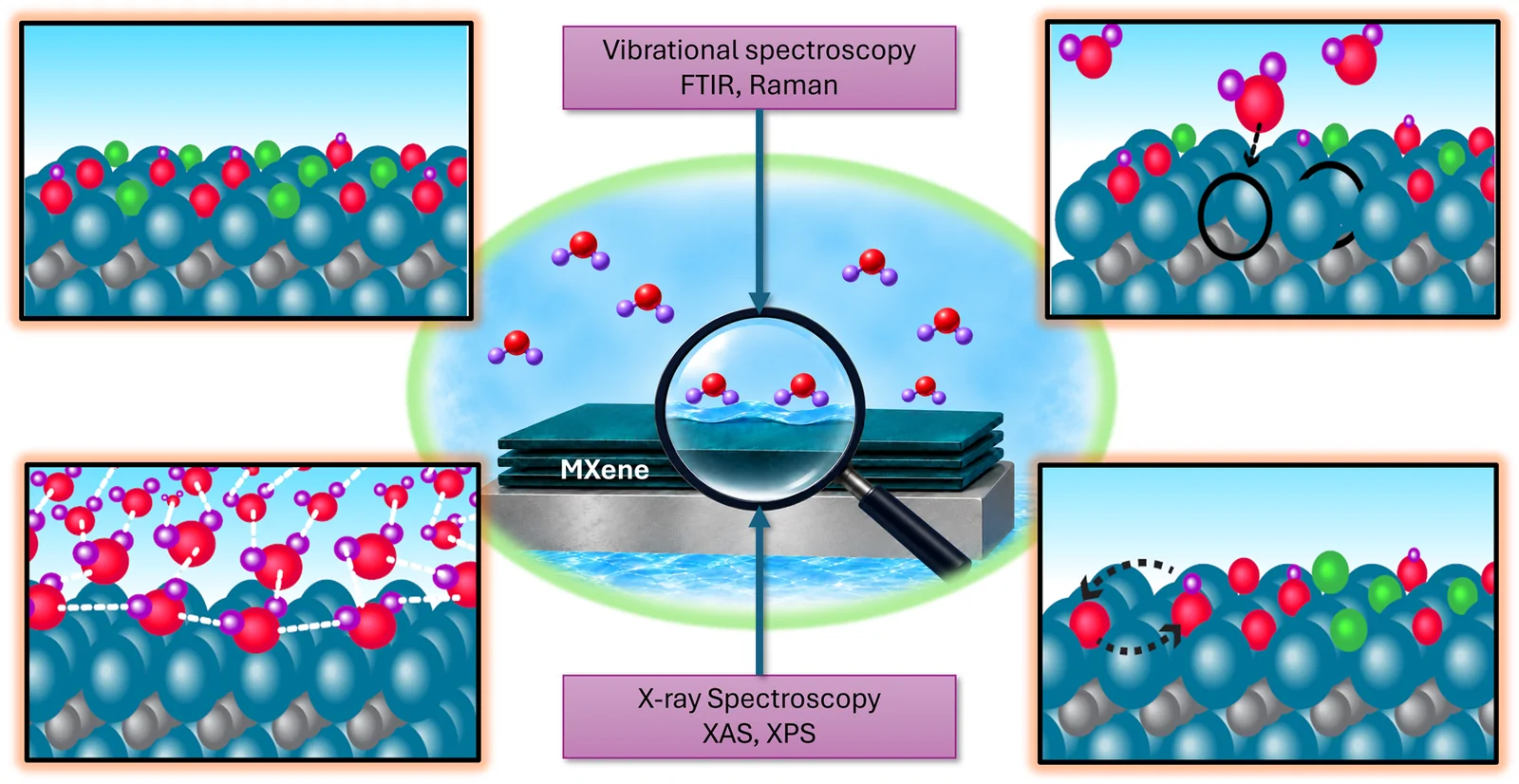

## 1 Introduction

Hydrogen enables renewable electricity to be stored as chemical energy and used when energy demand exceeds the immediate supply from intermittent renewable sources. It can also serve as a low-carbon feedstock and energy carrier in energy-intensive sectors where replacing fossil fuels *via* direct electrification remains particularly challenging. Among the available production routes, water electrolysis powered by renewable electricity provides a pathway for hydrogen production without direct carbon emissions during operation. The hydrogen evolution reaction (HER), which occurs at the cathode during water electrolysis, therefore plays a crucial role in sustainable hydrogen production. However, the efficiency of HER strongly depends on the electrocatalyst, and developing cost-effective, stable, and highly active materials remains an important challenge.

Platinum-group metal (PGM) catalysts are widely regarded as benchmark materials for the HER due to their near-optimal hydrogen adsorption energetics and high intrinsic activity.^1,2^ However, their high cost, scarcity, and limited scalability motivate the search for alternative, non-precious HER catalysts. Among emerging non-precious catalyst systems, MXenes have attracted growing attention alongside transition-metal dichalcogenides and other two-dimensional materials.^3^ MXenes are two-dimensional transition metal carbides and nitrides that are typically synthesized by selectively etching the A-layer from layered MAX phases. MAX phases are ternary carbides and nitrides with the general formula M_*n*+1_AX_*n*_, where M denotes an early transition metal, A is an element from groups 13–15, and X is carbon and/or nitrogen. These phases can exist in several structural forms, such as M_2_AX, M_3_AX_2_, and M_4_AX_3_.^4,5^ Selective removal of the A-layer yields two-dimensional MXene sheets with the general composition M_*n*+1_X_*n*_T_*x*_, where T_*x*_ represents surface terminations such as –O, –OH, and –F formed during synthesis.^6,7^ The broad compositional diversity of MAX precursors, together with the variety of possible surface terminations, results in a large family of MXenes with tunable chemical and physical properties (Fig. 1).^8^ This intrinsic structural and chemical tunability distinguishes MXenes from many conventional catalyst materials and suggests that their catalytic properties can, in principle, be tailored through materials design.

**Fig. 1:**
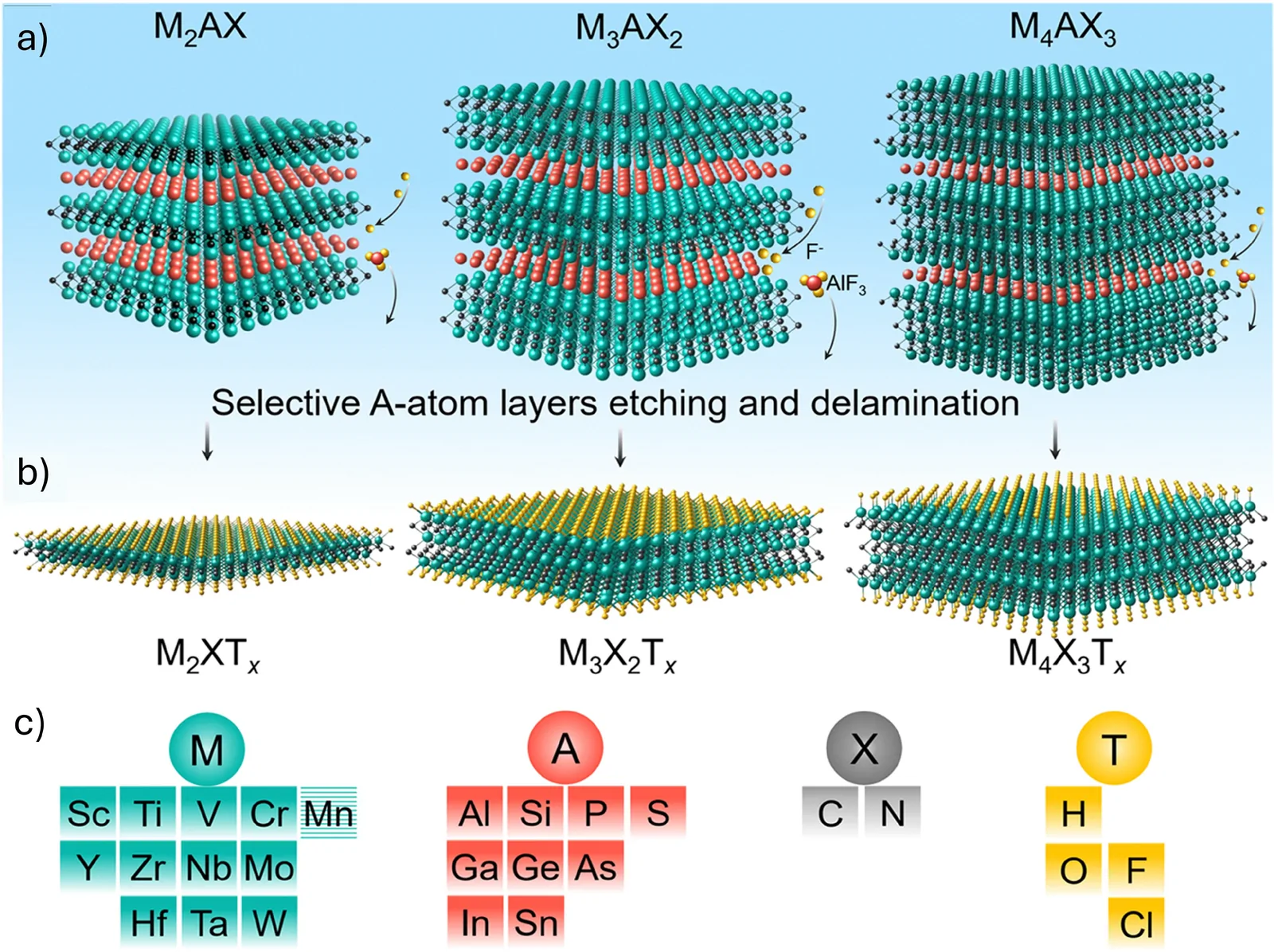
Schematic illustration of MXene structure and synthesis. (a) Representative MAX phase structures (M_2_AX, M_3_AX_2_, and M_4_AX_3_) and selective etching of the A-layer. (b) Formation of two-dimensional MXene sheets after etching and the development of surface terminations (T_*x*_) such as –O, –OH, and –F. (c) Examples of possible elements for M, A, X, and surface termination. Reproduced from ref. 8 with permission from Springer Nature, copyright 2020. This figure is excluded from the Creative Commons licence of this article.

A key manifestation of the chemical tunability of MXenes is the pronounced sensitivity of their electronic structure to surface termination chemistry. Theoretical studies have shown that identical MXene compositions can exhibit metallic or semiconducting behavior depending on the nature of the surface terminations, with substantial changes in band structure upon functionalization.^9^ For example, O-, OH-, and F-terminated MXenes with the same metal site have been predicted to display semiconducting behavior with distinct band gaps (Fig. 2a).^10^ Surface terminations also modulate the work function through termination-induced surface dipoles. In general, OH termination tends to lower the work function, O termination increases it, and F termination leads to intermediate or mixed behavior (Fig. 2b).^11^ Although F- and OH- are isoelectronic, their influence on the work function differs due to variations in electronegativity, bond polarity, and surface dipole orientation. OH-terminations typically generate a dipole oriented away from the MXene surface, lowering the vacuum level, whereas O and F terminations induce different charge redistributions that result in higher or intermediate work function values, respectively.^11,12^ These trends highlight that the electronic and interfacial properties of MXenes are not intrinsic material constants but are highly sensitive to surface chemistry, which is expected to play a central role in governing charge transfer and adsorption processes during electrocatalysis.

**Fig. 2:**
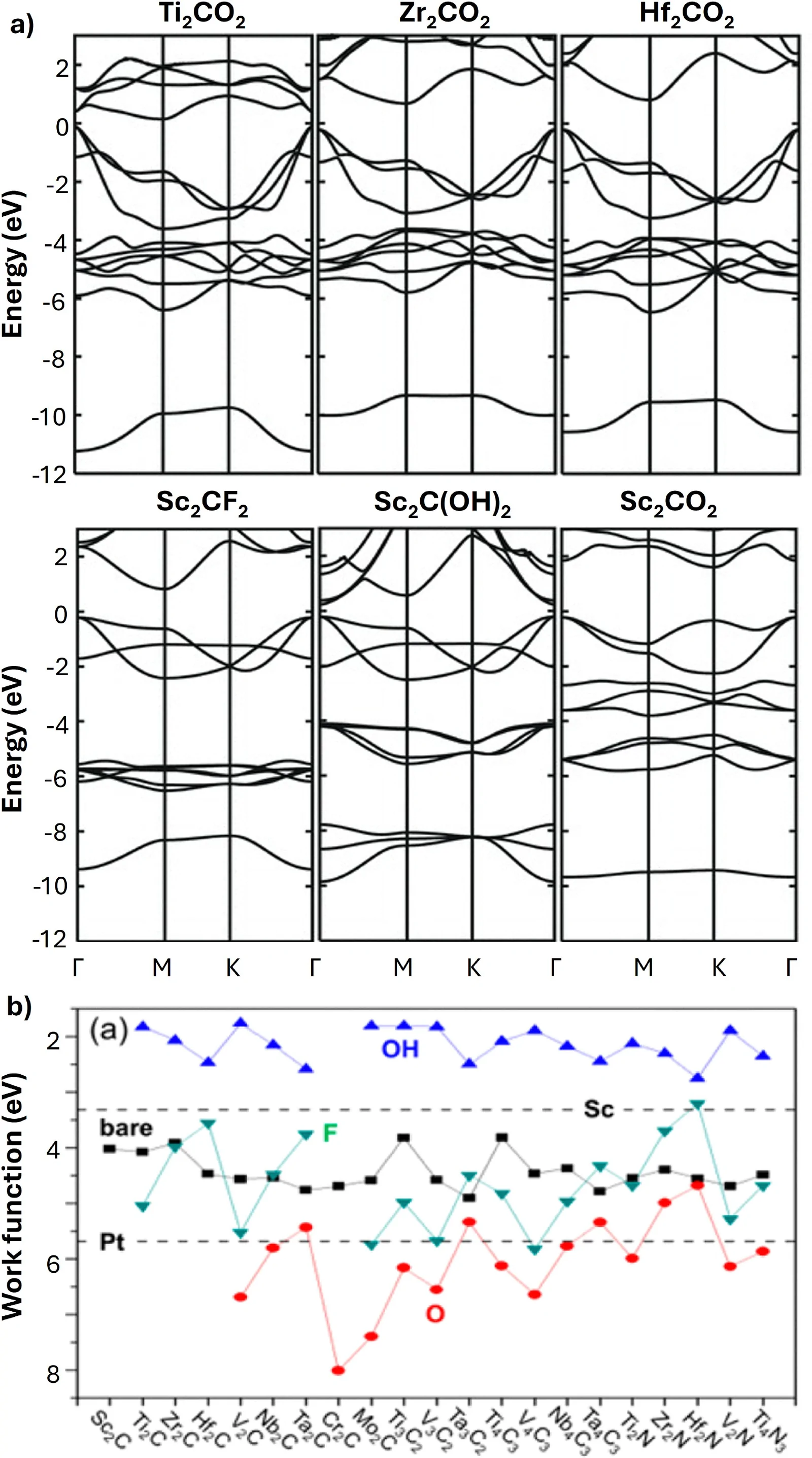
Electronic property tunability of MXenes *via* surface terminations. (a) Calculated band structures of selected MXenes with varying M or T sites (top and bottom panels, respectively), illustrating that surface functionalization (O, OH, F) can induce semiconducting behavior with different band gaps. Reproduced from ref. 10 with permission from Wiley-VCH, copyright 2013. (b) Work function variation of MXenes with different surface terminations, showing systematic trends depending on termination chemistry. Dashed lines indicate the work function of Sc and Pt for reference. Reprinted with permission from ref. 11. Copyright 2016 American Chemical Society.

To rationalize catalytic activity, many studies have proposed activity descriptors for HER on MXenes, including the classical view of optimized hydrogen adsorption free energy (Δ*G*_H_) based on the Sabatier principle, and materials properties connected to the bonding strength, like density of states near the Fermi level, work function, and surface termination composition. While these descriptors provide useful insights, they are often derived from idealized theoretical models or based on *ex situ* characterization of materials. In practice, MXene surfaces can undergo changes under electrochemical conditions due to reconstruction, oxidation, ion adsorption, or dynamic modification of surface terminations,^13–15^ similar to what has been observed for classical Pt catalysts^16,17^ and the anode counterparts such as transition metal oxides^18–20^ and hydroxides.^21^ As a result, there is often a gap between the assumed surface state used to define descriptors and the actual surface state during HER operation.

This discrepancy highlights the need for experimental approaches that can directly probe the active catalyst surface under realistic reaction conditions. *Operando* and *in situ* spectroscopic techniques, such as vibrational spectroscopies, X-ray absorption spectroscopy (XAS), and X-ray photoelectron spectroscopy (XPS), offer complementary insights into changes in adsorbates, oxidation states, surface chemistry, and electronic structure during electrochemical operation. Such techniques can therefore play a crucial role in validating, refining, or even revising proposed activity descriptors.

In this review, we discuss activity descriptors for HER on MXenes proposed from theoretical studies, together with experimentally observed activity trends. We highlight the challenges associated with probing the active MXene–electrolyte interface and summarize recent progress in applying *operando* spectroscopic techniques to MXene systems and related materials. Finally, we discuss how *operando* insights can contribute to a more reliable understanding of structure–property–activity relationships and support the rational design of MXene-based electrocatalysts.

## 2 MXenes as HER electrocatalysts

MXenes have attracted increasing attention as potential electrocatalysts for the hydrogen evolution reaction because of several favorable properties. Their two-dimensional structure provides a high density of accessible surface sites, and many MXene compositions show metallic conductivity, which supports efficient charge transport during electrocatalysis. In addition, the surface termination strongly influences the adsorption behavior and surface reaction energetics. Interestingly, the surface chemistry of MXenes is not fixed and can change depending on synthesis conditions and post-treatments. This additional degree of tunability is particularly relevant for HER.

Over the past few years, an increasing number of studies have investigated HER on MXenes using both computational and experimental approaches. The reported activity trends vary widely and are often sensitive to composition and surface chemistry. In this section, we summarize the current understanding of HER activity on MXenes. We first discuss theoretical predictions, then review experimental observations, and finally describe activity descriptors proposed in the literature. This overview provides the basis for the later discussion in this review.

### 2.1 Theoretical insights into HER activity on MXenes

Density functional theory (DFT) calculations have been widely used to screen the potential HER activity of MXenes by evaluating the hydrogen adsorption free energy (Δ*G*_H_),^22,23^ which is commonly employed as a first-order descriptor for HER activity.^1^ Within this framework, optimal catalytic behavior is expected when Δ*G*_H_ is close to thermoneutral, while significantly positive or negative values indicate weak or strong hydrogen binding, respectively. Based on this concept, several computational studies have reported so-called HER volcano plots for different carbide- and nitride-based MXenes. Such volcano plots relate HER activity to bond strength (or related descriptors), forming a volcano-shaped curve with the Sabatier optimum at its peak, where hydrogen binding is neither too strong nor too weak. It should be noted, however, that most of these screening studies rely on GGA-level functionals without explicit on-site Hubbard corrections. For MXenes containing transition metals with partially filled 3d states, localized d-electron interactions may influence the electronic structure near the Fermi level and, consequently, the predicted adsorption energetics. While recent studies have begun to explore DFT+*U* treatments for selected MXene compositions,^24,25^ systematic comparisons of HER trends obtained with and without Hubbard corrections remain limited. Therefore, reported Δ*G*_H_ trends should primarily be interpreted in a comparative rather than absolute sense.

A representative screening study examined a broad set of M_2_X MXenes (M = Sc, Ti, Zr, Hf, V, Nb, Ta, Cr, Mo, and W; X = C or N), with surface terminations predicted based on their relative thermodynamic stability under HER conditions.^23^ The resulting volcano plot (Fig. 3a) shows a wide spread in the predicted HER activity. Most MXenes are located on the weak-binding side of the volcano, which indicates generally weak hydrogen adsorption on the basal planes. However, some compositions are positioned closer to the volcano peak and therefore appear more promising for HER.

**Fig. 3:**
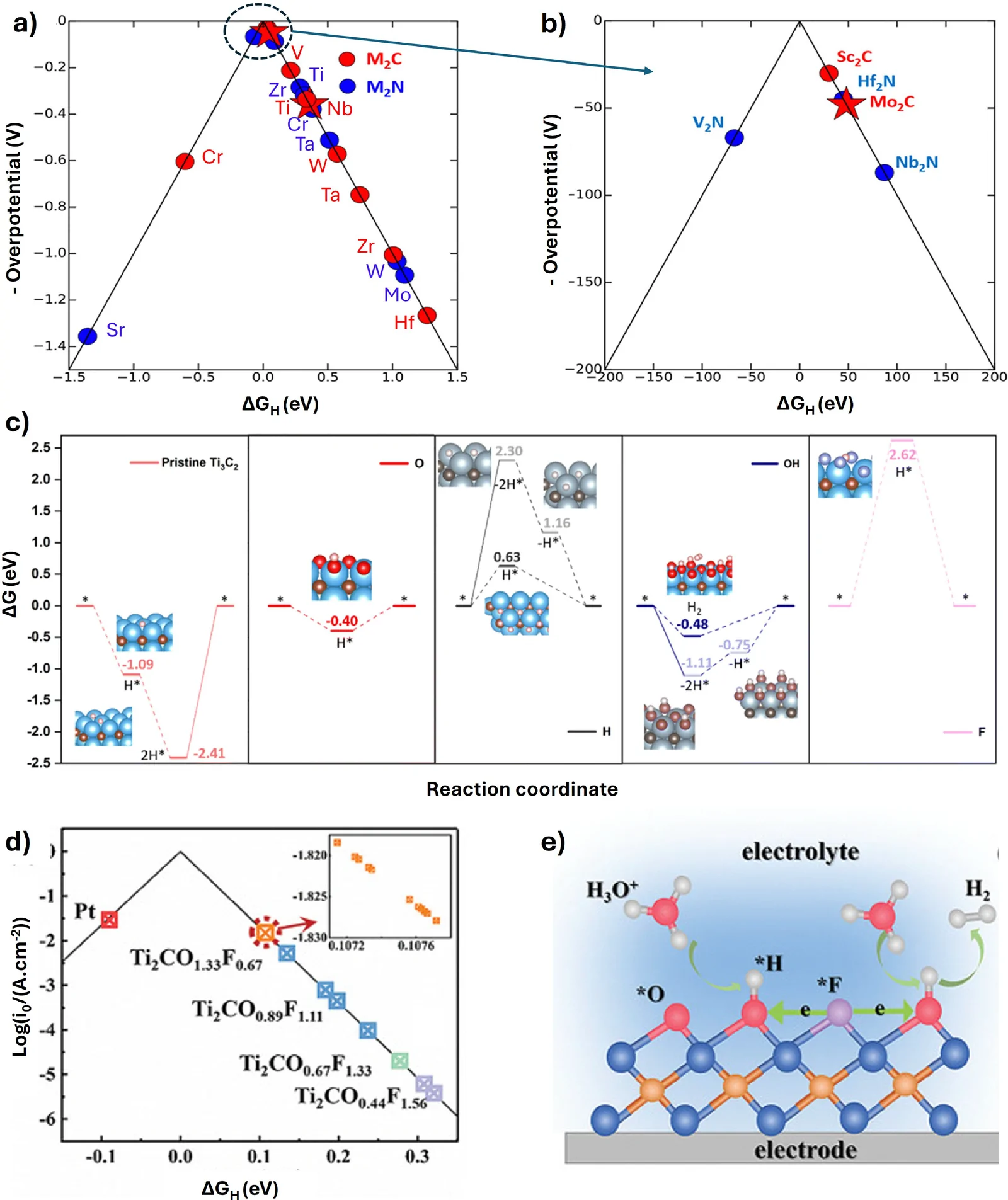
Theoretical insights into HER activity on MXenes. (a) DFT-derived HER volcano plot relating hydrogen adsorption free energy (Δ*G*_H_) to theoretical overpotential for a broad range of M_2_C and M_2_N MXenes. (b) Magnified view of the top region of the volcano highlighting promising compositions with low predicted overpotentials. (c) Gibbs free energy (Δ*G*) reaction diagrams for HER on Ti_3_C_2_T_*x*_ surfaces with different terminations, demonstrating strong hydrogen binding on pristine surfaces and weak binding on F-terminated surfaces. Solid lines denote chemical H_2_ desorption, and dashed lines represent electrochemical steps of proton-coupled electron transfer (PCET). Atom colors: Ti (blue), C (brown), O (red), H (white), and F (violet). (d) Termination-dependent volcano relationship for Ti_2_CT_*x*_ with varying O/F ratios, showing improved activity with increasing O content. (e) Schematic illustration of the HER mechanism on Ti_2_CT_*x*_ in acidic media, indicating preferential hydrogen adsorption on O-terminated sites. Panels (a) and (b) adapted with permission from ref. 23, Copyright 2016 American Chemical Society. Panel (c) reproduced from ref. 26 with permission from the Royal Society of Chemistry, copyright 2023; permission conveyed through Copyright Clearance Center, Inc. Panels (d) and (e) reproduced from ref. 27 with permission from Wiley-VCH, copyright 2021.

A magnified view of the top region of the volcano (Fig. 3b) highlights several candidates with theoretically low overpotentials. These include functionalized Sc_2_CT_*x*_ and Mo_2_CT_*x*_ among the carbides, and V_2_NT_*x*_, Hf_2_NT_*x*_, and Nb_2_NT_*x*_ among the nitrides. These results indicate that the choice of transition metal and the carbide or nitride nature of the MXene both have a strong influence on the predicted HER activity. The influence of the transition metal has been associated, in part, with differences in work function, which shift the Fermi level relative to the vacuum level and can thereby modify charge transfer to adsorbed hydrogen and its adsorption energetics. Such screening studies do not guarantee that all predicted materials can be synthesized or remain stable under experimental conditions. Instead, they provide a useful starting point for identifying promising compositions and for interpreting observed activity trends.

Volcano plots provide a convenient thermodynamic screening metric for comparing a large number of materials. However, they do not explicitly resolve the sequence of elementary steps or the detailed reaction pathway. To address this, reaction coordinate analyses based on Gibbs free energy diagrams have been used to examine the HER mechanism on specific MXene surfaces with different terminations.^26^ Beyond broad materials screening, such DFT-based free energy calculations allow a direct assessment of how surface termination chemistry modifies individual reaction steps. Fig. 3c illustrates the calculated HER free energy profiles for pristine and fully O-, OH-, F-, and H-terminated Ti_3_C_2_ surfaces under standard conditions. A clear dependence of reaction energetics on surface termination is observed. Pristine Ti_3_C_2_ binds hydrogen too strongly, leading to highly unfavorable hydrogen recombination despite downhill proton–electron transfer steps. In contrast, F-terminated surfaces exhibit extremely weak hydrogen affinity, making the initial proton adsorption step energetically unfavorable. These two limiting cases highlight that both overly strong and overly weak adsorption are detrimental to HER activity. More balanced behaviour is found for O- and OH-terminated surfaces. Importantly, these results emphasize that HER activity predictions based solely on pristine surfaces can be misleading, and that realistic surface chemistries must be considered when linking theoretical predictions to experimentally observed behavior.

The influence of surface terminations on HER activity is directly reflected in termination-dependent activity trends. Fig. 3d presents a volcano-type relationship between predicted exchange current density and Δ*G*_H_ for Ti_2_CT_*x*_ models with varying O/F ratios.^27^ A clear trend emerges in which increasing the fraction of O terminations systematically shifts the material closer to the top of the volcano, whereas increasing F content drives the system toward poorer activity on the weak-binding side. Among the investigated compositions, Ti_2_CO_1.33_F_0.67_, possessing the highest O content, is predicted to exhibit the most favorable HER performance, while F-rich compositions show substantially reduced activity. These results further indicate that increasing F content can be detrimental to HER activity and underscore the importance of surface termination chemistry as a key design parameter.

At the atomistic level, this behavior can be rationalized by examining the elementary reaction steps on differently terminated surfaces. As illustrated schematically in Fig. 3e, hydrogen adsorption and subsequent proton–electron transfer steps preferentially occur at O- and OH-terminated sites when such species are present on the basal plane.^27^ These terminations can chemisorb protons and transiently form surface-bound –OH_2_^+^-like intermediates that facilitate proton transfer and hydrogen evolution. In contrast, F-terminated regions contribute little to catalytic activity, as the high electronegativity of fluorine limits proton interaction to weak electrostatic attraction rather than the formation of strongly bound reaction intermediates. This mechanistic picture highlights that MXene surfaces typically consist of a heterogeneous distribution of active (O/OH-terminated) and inactive (F-terminated) sites, and that the overall HER performance is governed by the relative abundance and spatial distribution of these terminations rather than by bulk composition alone.

### 2.2 Experimental insights into HER activity

Experimental studies show that the HER performance of MXenes can be tuned at several levels, ranging from the choice of MXene composition and synthesis-dependent surface chemistry to deliberate post-synthetic modification and the construction of heterointerfaces. These factors influence not only the measured catalytic activity but also its retention as the material undergoes chemical or structural changes. Representative examples of these experimentally accessible strategies are summarized in Fig. 4.

**Fig. 4:**
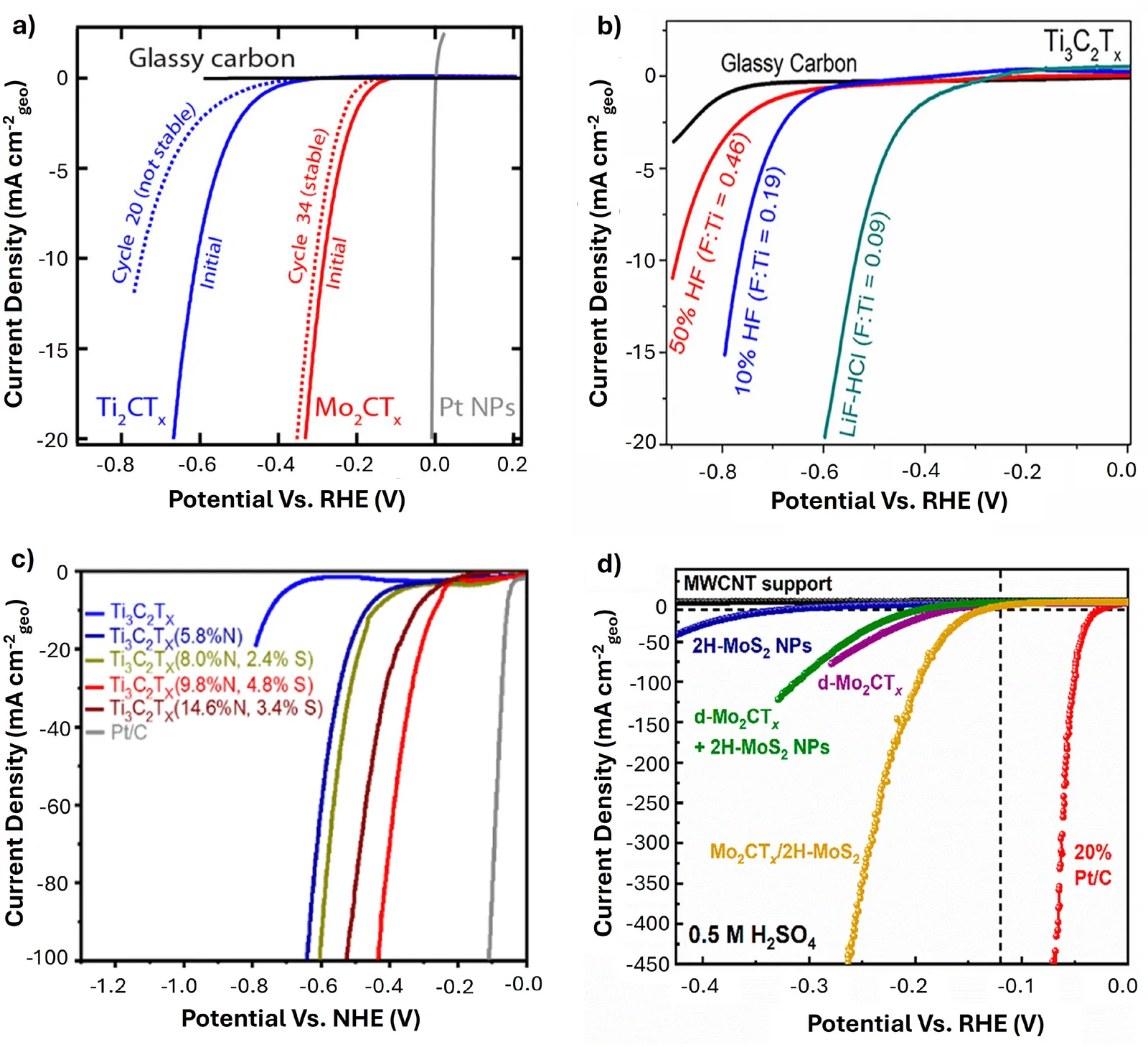
Experimental insights into HER activity of MXenes. (a) LSV comparison showing higher HER activity of Mo_2_CT_*x*_ compared to Ti_2_CT_*x*_. (b) Effect of synthesis route and surface chemistry on HER activity of Ti_3_C_2_T_*x*_. (c) Effect of N/S anion engineering on the HER polarization behavior of Ti_3_C_2_T_*x*_. (d) HER polarization curves comparing Mo_2_CT_*x*_/2H-MoS_2_ with the individual components and their physical mixture. Panel (a) reproduced with permission from ref. 23, Copyright 2016 American Chemical Society. Panel (b) reproduced with permission from ref. 28, Copyright 2018 American Chemical Society. Panel (c) reproduced from ref. 29 with permission from the American Chemical Society, copyright 2023; permission conveyed through Copyright Clearance Center, Inc. Panel (d) reproduced from ref. 30 with permission from the American Chemical Society, copyright 2020; permission conveyed through Copyright Clearance Center, Inc.

Fig. 4a compares the HER activity of Ti_2_CT_*x*_ and Mo_2_CT_*x*_.^23^ Ti_2_CT_*x*_ shows measurable activity but requires a relatively high overpotential to reach 10 mA cm^−2^ and its performance decreases upon repeated cycling. In contrast, Mo_2_CT_*x*_ exhibits much higher activity, achieving the same current density at significantly lower overpotential and maintaining stable performance after initial cycling. This experimental trend is consistent with the theoretical screening discussed in Section 2.1, in which the corresponding Mo-based composition exhibited more favorable hydrogen adsorption energetics than its Ti-based counterpart. These results confirm that MXenes can act as HER electrocatalysts and that their activity strongly depends on composition. The different responses upon cycling further indicate that MXene composition can influence not only initial HER activity but also its retention during electrochemical operation.

The strong influence of surface termination chemistry on HER activity is clearly demonstrated by experiments on Ti_3_C_2_T_*x*_ synthesized using different etching routes (Fig. 4b).^28^ In this work, Ti_3_C_2_T_*x*_ was prepared using LiF–HCl, 10% HF, and 50% HF etching conditions. The underlying Ti_3_C_2_ framework remains identical in all cases; however, the etching chemistry systematically modifies the surface termination composition. The central idea is to generate MXenes with comparable structure and morphology but different ratios of –O, –OH, and –F terminations. Linear sweep voltammetry shows that the HER activity depends on the etching route used to synthesize the MXene, following the trend LiF–HCl > 10% HF > 50% HF, with the LiF–HCl etched sample exhibiting the lowest overpotential. Since these samples have similar electrochemical surface areas, the observed differences cannot be explained by surface area effects alone. Instead, XPS analysis reveals that the F : Ti ratio increases in the order LiF–HCl < 10% HF < 50% HF, indicating that higher fluorine content leads to poorer HER performance. These results directly support the theoretical prediction that fluorine terminations weaken hydrogen adsorption and reduce catalytic activity. Furthermore, partial removal of fluorine terminations through alkaline treatment significantly improves catalytic performance, highlighting the importance of surface termination engineering for optimizing MXene electrocatalysts for HER.^28^ Overall, these experimental examples illustrate why reported HER performance of MXenes can vary widely across the literature. Differences in synthesis routes and post-treatments often lead to different surface terminations and surface states, which in turn strongly affect the measured activity.

Importantly, this surface chemistry can also be deliberately modified after MXene synthesis. Tiwari *et al.* used N/S anion engineering of Ti_3_C_2_T_*x*_, producing Ti–N and Ti–S bonding while simultaneously modifying the native O- and F-containing surface chemistry.^29^ As shown in Fig. 4c, the optimized N/S-modified sample exhibits a pronounced improvement in HER activity relative to pristine Ti_3_C_2_T_*x*_. The improvement remained evident after normalization to the electrochemically active surface area and was accompanied by lower charge-transfer resistance, while DFT calculations showed more favorable hydrogen adsorption energetics for the co-modified surface.^29^ Similar sensitivity to post-synthetic modification has been observed upon low-temperature annealing of Ti_3_C_2_T_*x*_, where changes in Ti–O, –OH, and –F surface species were accompanied by substantial changes in HER activity.^31^ More recently, N and Co incorporation has also been shown to modify the HER response of Ti_3_C_2_T_*x*_.^32^ Collectively, these studies demonstrate that post-synthetic chemical modification provides an additional route to tune MXene electrocatalysis. At the same time, such treatments can concurrently alter termination chemistry, local bonding, accessible surface area, interlayer structure, and charge-transfer properties, making it difficult to attribute the resulting activity enhancement to a single structural parameter.

The connection between structural evolution and catalytic performance becomes particularly important when stability is considered. Sunderiya *et al.* followed the progressive aqueous oxidation of Ti_3_C_2_T_*x*_ and observed a non-monotonic HER response: the overpotential at 10 mA cm^−2^ increased from 0.435 to 0.877 V after three weeks of aging, but subsequently decreased to approximately 0.40 V after more extensive oxidation.^33^ This evolution was accompanied by progressive loss of Ti–C bonding and formation of oxidized Ti species, with substantial transformation of the original carbide structure upon prolonged oxidation. The initial deterioration in HER activity was associated with reduced conductivity, whereas calculations indicated that further oxygen incorporation could shift hydrogen adsorption energetics back toward more favorable values.^33^ This example highlights an important distinction between catalytic and structural stability: retention or recovery of HER activity does not necessarily imply preservation of the original MXene structure.

Heterointerface engineering provides another route to modify both catalytic activity and durability. Lim *et al.* converted surface oxide on Mo_2_CT_*x*_ into epitaxially coupled 2H-MoS_2_, producing a Mo_2_CT_*x*_/2H-MoS_2_ nanohybrid (Fig. 4d).^30^ The nanohybrid required an overpotential of only 119 mV to reach 10 mA cm^−2^ in 0.5 M H_2_SO_4_ and outperformed untreated Mo_2_CT_*x*_, 2H-MoS_2_, and their physical mixture. The enhanced performance was associated with the intimate Mo_2_CT_*x*_/2H-MoS_2_ interface together with the removal of detrimental MoO_3−*x*_ species and improved interfacial charge transfer.^30^ Importantly, the same architecture also suppressed further oxidation of the MXene, with an increase in overpotential of less than 30 mV after 10 days of continuous electrolysis or 100 000 cyclic voltammetry cycles, whereas untreated Mo_2_CT_*x*_ showed pronounced oxidation and performance loss.^30^ This provides a direct example in which interface design links structural stabilization with improved activity retention.

Recent studies extend this strategy to a broader range of MXene-based hybrid interfaces. Chemical bonding between Ti_3_C_2_T_*x*_ and the two-dimensional Ni_3_(HITP)_2_ metal–organic framework has been shown to improve HER activity through interfacial Ti–N bonding and associated changes in charge transfer and electrochemically accessible active area.^34^ Double-transition-metal MXenes have likewise been combined with CoS_2_ and MoS_2_, with Mo_2_Ti_2_C_3_T_*x*_-based hybrids showing enhanced alkaline HER performance and sustained operation.^35,36^ More broadly, integrating components of different dimensionalities with MXenes provides a strategy for controlling interlayer architecture and functional interfaces, with potential implications for electrocatalytic performance.^37^ These studies illustrate how the experimentally accessible design space extends beyond the chemistry of an isolated MXene surface to interactions created between MXenes and secondary catalytic phases.

Taken together, the experimental literature establishes a close relationship between MXene structure, catalytic activity, and stability. Composition and synthesis-dependent surface chemistry determine the initial catalytic properties, while post-synthetic modification and heterointerface formation can further alter adsorption energetics, accessible active sites, charge transfer, and resistance to degradation. However, the same studies also show that changes in catalytic performance can accompany substantial chemical or structural evolution of the material. Consequently, activity and stability measurements alone do not identify which surface or structural state is responsible for the observed HER response. This distinction becomes particularly important when relating experimentally measured HER performance to proposed activity descriptors and, ultimately, to the active MXene interface under electrochemical conditions.

### 2.3 Beyond Δ*G*_H_: proposed descriptors for HER activity on MXenes

An appropriate activity descriptor can be used to efficiently screen catalyst candidates with potentially high HER activity. As mentioned in the preceding section, the hydrogen adsorption free energy (Δ*G*_H_) is the most widely used descriptor, with ideal catalysts expected to exhibit values close to zero. However, Δ*G*_H_ represents a thermodynamic measure of hydrogen binding strength that is not directly accessible experimentally and can be sensitive to the computational treatment of the MXene surface. For this reason, several additional descriptors have been proposed to better understand the underlying factors that control Δ*G*_H_ and, in turn, HER activity. In most cases, these descriptors are validated *via* robust scaling with calculated Δ*G*_H_ values, suggesting that they capture important aspects of surface chemistry, electronic structure, or local bonding environment. Beyond providing physical insight, such descriptors are also attractive because they can, in some cases, be accessed experimentally or inferred from spectroscopic observables, thereby enabling closer integration between theory and experiment. These descriptors therefore do not necessarily replace Δ*G*_H_, but complement it by providing additional physical insight into why certain MXenes exhibit more favorable HER behavior than others.

#### 2.3.1 Number of electrons gained by surface oxygen (*N*_e_)

For O-terminated MXenes, the intrinsic HER activity of the basal plane is governed by the strength of the surface O–H bond, which in turn is determined by the electronic structure of the surface oxygen species. From a molecular orbital perspective, adsorption of H on an O-terminated surface involves the interaction between the H 1s orbital and the O 2p_*z*_ orbital, leading to the formation of a low-energy bonding (σ) orbital and a high-energy antibonding (σ*) orbital (Fig. 5a).^38^ The occupancy of the antibonding σ* state plays a central role in determining adsorption strength: higher σ* occupancy weakens the O–H bond and results in less negative (or even positive) hydrogen adsorption free energies, whereas low σ* occupancy leads to overly strong binding.

**Fig. 5:**
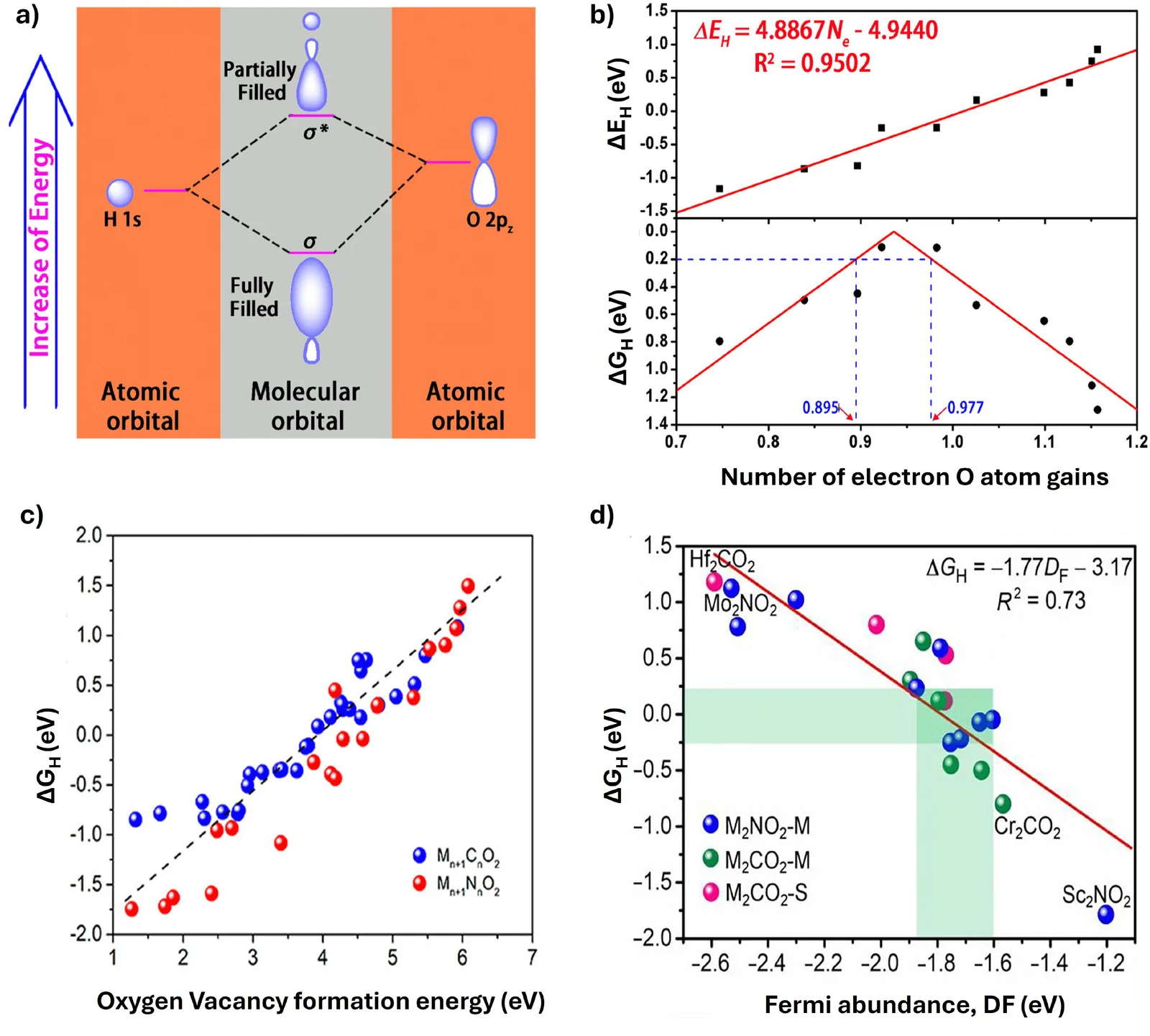
Overview of proposed activity descriptors for HER on MXenes. (a) Schematic molecular orbital interaction between H 1s and surface O 2p_*z*_ orbitals on O-terminated MXenes, illustrating the formation of bonding (σ) and antibonding (σ*) states that govern H–O bond strength; (b) correlation between the number of electrons gained by surface oxygen (*N*_e_) and hydrogen adsorption energy; (c) relationship between oxygen vacancy formation energy and Δ*G*_H_ for oxygen-terminated MXenes, including both carbide and nitride systems; (d) relationship between Fermi abundance (*D*_F_) and Δ*G*_H_; panels (a) and (b) reprinted with permission from ref. 38, Copyright 2016 American Chemical Society. Panel (c) adapted with permission from ref. 39, Copyright 2018 American Chemical Society. Panel (d) reprinted from Science Bulletin, vol. 63, X. Bai *et al.*, “Insight into the catalytic activity of MXenes for hydrogen evolution reaction”, pp. 1397–1403, Copyright 2018, with permission from Elsevier.^40^

As the degree of charge transfer from the underlying transition metal to surface oxygen depends on the metal's electron-donating ability, the electronic population of surface O varies systematically across different MXenes. On this basis, Ling *et al.* proposed the number of electrons gained by surface oxygen (*N*_e_), as determined through DFT-based charge analysis of the transition metal–oxygen bond, as a physically meaningful activity descriptor.^38^ This quantity directly reflects the filling of O-derived electronic states and therefore encodes the strength of the O–H interaction. Using a set of fully O-terminated M_2_CO_2_ MXenes, a strong linear correlation was observed between *N*_e_ and the hydrogen adsorption energy, indicating that *N*_e_ captures the same chemical physics that ultimately governs Δ*G*_H_ (Fig. 5b). When the absolute value of Δ*G*_H_ is plotted as a function of *N*_e_, a volcano-type relationship emerges, with an optimal window of *N*_e_ values associated with near-thermoneutral hydrogen adsorption.

Importantly, this descriptor provides practical design insight rather than only a correlation. Because *N*_e_ reflects the extent of electron donation from the metal layers to surface oxygen, it suggests that HER activity can be tuned through compositional engineering. For example, introducing a second metal with a different electron-donating ability can adjust the charge density on surface O and shift the system toward the optimal *N*_e_ range.^41^ In this way, *N*_e_ functions as an electronic–structure–informed descriptor that complements Δ*G*_H_ and helps rationalize compositional trends in HER activity across O-terminated MXenes, analogous to the d-band model developed for metal-based electrocatalysis^42^ and to electronic–structure descriptors used in oxygen evolution catalysis on transition-metal oxides, where the occupancy of antibonding σ* (e_g_) orbitals has been correlated with activity.^43,44^

#### 2.3.2 Oxygen vacancy formation energy

Jiang *et al.* have shown that previously proposed descriptors such as the lowest unoccupied state of surface oxygen (*ε*_LUS_) and the number of electrons gained by oxygen (*N*_e_) lose predictive power when applied to a broader family of MXenes beyond simple M_2_CO_2_ systems.^39^ They therefore proposed the oxygen vacancy formation energy as a more general descriptor for HER activity on O-terminated MXenes. Oxygen vacancy formation energy correlates with the strength of the transition-metal–oxygen bond, which also influences the electron transfer discussed in the context of *N*_e_. It is further motivated by the observation that, upon hydrogen adsorption, the surface O atom does not remain rigid but tends to protrude from the basal plane, inducing local lattice distortion. A meaningful descriptor should therefore capture not only electronic effects but also the energy associated with structural relaxation. This parameter reflects the strength of the O–metal interaction and implicitly includes the energetic contribution of local distortion, making it more physically representative of the adsorption process.

In Fig. 5c a strong linear relationship between oxygen vacancy formation and Δ*G*_H_ is observed across a wide range of oxygen-terminated carbide MXenes, with a high coefficient of determination (*R*^2^ ≈ 0.88), indicating that it can reliably predict HER trends within this family. Importantly, this scaling is not limited to carbides. When extended to oxygen-terminated nitride MXenes, a similarly strong correlation is preserved (*R*^2^ ≈ 0.94), suggesting that oxygen vacancy formation energy may provide a more transferable descriptor across different MXene chemistries and layer thicknesses.^39^

From a practical perspective, this descriptor offers a computationally efficient way to screen large numbers of candidate MXenes and to identify materials expected to exhibit near-optimal hydrogen adsorption. At the same time, its formulation remains grounded in the physical chemistry of the interface, as it captures the coupling between hydrogen adsorption, lattice response, and surface bonding. A limitation, however, is that the calculated vacancy formation energy and its correlation with HER activity may depend on hydrogen coverage and surface configuration, especially in the presence of potential-dependent mixed surface terminations. This suggests that its predictive power should be interpreted with caution under realistic, dynamic operating environments.

#### 2.3.3 Fermi-abundance descriptor (*D*_F_)

To further link intrinsic catalytic activity to electronic structure, Bai *et al.* proposed the Fermi-abundance (*D*_F_) model to capture how the electronic states close to the Fermi level (*E*_F_) control the intrinsic HER activity of MXenes.^40^ The key idea is that not all electronic states contribute equally to surface reactivity; instead, states located near the Fermi level are most relevant for bonding with reaction intermediates. The *D*_F_ descriptor therefore quantifies the abundance of occupied electronic states near *E*_F_ by weighting the density of states around the Fermi level, providing a physically intuitive measure of how strongly the surface can interact with adsorbed hydrogen.^45^

Using this approach, the authors examined a set of 20 O-terminated MXenes (M_2_CO_2_ and M_2_NO_2_) and observed an approximately linear relationship between Δ*G*_H_ and *D*_F_ when an appropriate weighting parameter was used (Fig. 5d).^40^ A larger magnitude of *D*_F_ corresponds to a higher density of occupied states near the Fermi level and thus stronger H binding, whereas a smaller magnitude reflects weaker interaction. Importantly, an optimal window of *D*_F_ values between approximately −1.60 and −1.87 eV was identified, within which several MXenes exhibit near-thermoneutral hydrogen adsorption and are therefore predicted to be intrinsically active for HER. Unlike descriptors based on single parameters such as charge transfer or bond length, *D*_F_ directly reflects the electronic structure of the active surface and offers a conceptually closer link to the origin of catalytic activity.

In addition to the descriptors discussed above, other electronic and structural parameters have been proposed to rationalize HER activity trends on MXenes. For example, the electronic–structural descriptor *ϕ* combines information from the Fermi level and the local metal–oxygen bonding environment to estimate how strongly hydrogen interacts with O-terminated MXene surfaces.^46–48^ Similarly, the position of the O 2p band center has been suggested as an indicator of the reactivity of surface oxygen atoms, reflecting how the electronic states of the termination participate in hydrogen adsorption.^49^ A comparative overview of these HER activity descriptors, including their physical basis, calculation methods, experimental accessibility, strengths, and limitations, is provided in Table 1.

**Table 1:** Comparison of theoretical descriptors proposed for HER activity in MXenes

Descriptor	Physical meaning	Calculation method	Experimental access	Strength	Limitation
Hydrogen adsorption free energy (Δ*G*_H_)^1,22,23^	Free-energy change for H adsorption; reflects H–surface binding strength	DFT adsorption energies with zero-point-energy and entropy corrections	Not directly measurable; indirectly assessed through HER activity	Directly linked to the Sabatier principle and widely used for HER screening	Sensitive to adsorption site, surface termination, H coverage, and model environment
Number of electrons gained by surface oxygen (*N*_e_)^38^	Charge gained by surface O; reflects charge transfer and O–H bonding	DFT-based charge analysis	Not directly measurable; related charge redistribution can be probed spectroscopically	Simple electronic descriptor linking surface–O charge to H adsorption	Developed mainly for O-terminated MXenes; correlation with Δ*G*_H_ can vary with H coverage
Oxygen-vacancy formation energy^39^	Energy required to form a surface O vacancy; reflects O binding and local structural relaxation	DFT total-energy calculations for pristine and O-vacancy structures	O-vacancy-related structural changes can be probed experimentally, but the formation energy is not directly measurable	Combines electronic and structural contributions relevant to H adsorption	Primarily established for O-terminated MXenes and may depend on H coverage and surface configuration
Fermi abundance (*D*_F_)^40^	Abundance of electronic states near the Fermi level; reflects states relevant to H–surface interaction	Electronic–structure and density-of-states calculations	Near-Fermi electronic states can be probed spectroscopically	Links near-Fermi electronic structure with H adsorption	Does not explicitly account for surface geometry or identify the active configuration
Electronic–structural descriptor (*ϕ*)^46–48^	Combines Fermi-level and local metal–oxygen bonding information relevant to H adsorption	Electronic–structure calculations combined with local structural parameters	Underlying electronic and structural quantities can be probed indirectly	Captures coupled electronic and structural contributions in a single descriptor	Demonstrated mainly for idealized O-terminated MXenes; broader transferability remains to be established
O 2p-band center (*ε*_p_)^49^	Energy center of surface–O 2p states; reflects O–MXene and O–H electronic interactions	DFT projected density-of-states analysis	O-derived electronic states can be probed spectroscopically, although *ε*_p_ is generally obtained computationally	Links the electronic structure of surface O with H adsorption strength	Established mainly for idealized O-terminated carbide MXenes; applicability to mixed terminations remains uncertain

Across the literature, several descriptors have been proposed to explain and predict HER activity trends on MXenes, and many of them show encouraging correlations with calculated Δ*G*_H_ within their respective datasets. However, these descriptors are largely developed from idealized surface models and density functional theory studies, often assuming uniform terminations, well-defined structures, and simplified reaction environments. As a result, their broader reliability remains unclear when applied to real electrodes, where surface chemistry, defects, mixed terminations, electrolyte effects, and potential-dependent changes can strongly influence the active interface. Importantly, many of the parameters underlying these descriptors—such as electronic states near the Fermi level, band positions, and surface bonding environments—are directly related to quantities that can in principle be probed by spectroscopic techniques. This gap between theoretical descriptors and realistic operating conditions therefore highlights the need for experimental approaches that can directly track surface composition and electronic structure during HER.

## 3 Importance of probing the active interface

Understanding the catalytic behavior of MXenes in the HER requires accurate identification of the active interface under operating conditions. In electrochemical systems, the catalyst–electrolyte interface is governed by multiple interdependent factors, including applied potential, electrolyte composition, pH, solvent organization, and interfacial charge redistribution. These factors collectively define the local reaction environment and determine how the catalyst interacts with reactants during operation. This section therefore focuses on the key physicochemical processes that shape the active interface under realistic electrochemical conditions. Importantly, identifying this interface also requires distinguishing structural changes that generate or preserve an active catalytic state from those that progressively degrade the catalyst during operation.

### 3.1 MXene surfaces evolve under electrochemical conditions

MXene surfaces can undergo structural and chemical evolution under electrochemical conditions through dynamic interactions with the electrolyte, thereby redefining the nature of the active interface during HER. Variations in applied potential and solvent structure can induce changes in surface terminations, local atomic arrangements, and interfacial bonding. Processes such as partial oxidation, defect formation, and dissolution can also occur, particularly at reactive sites. The MXene–electrolyte interface should therefore be regarded as a dynamically evolving system, as schematically illustrated in Fig. 6.

**Fig. 6:**
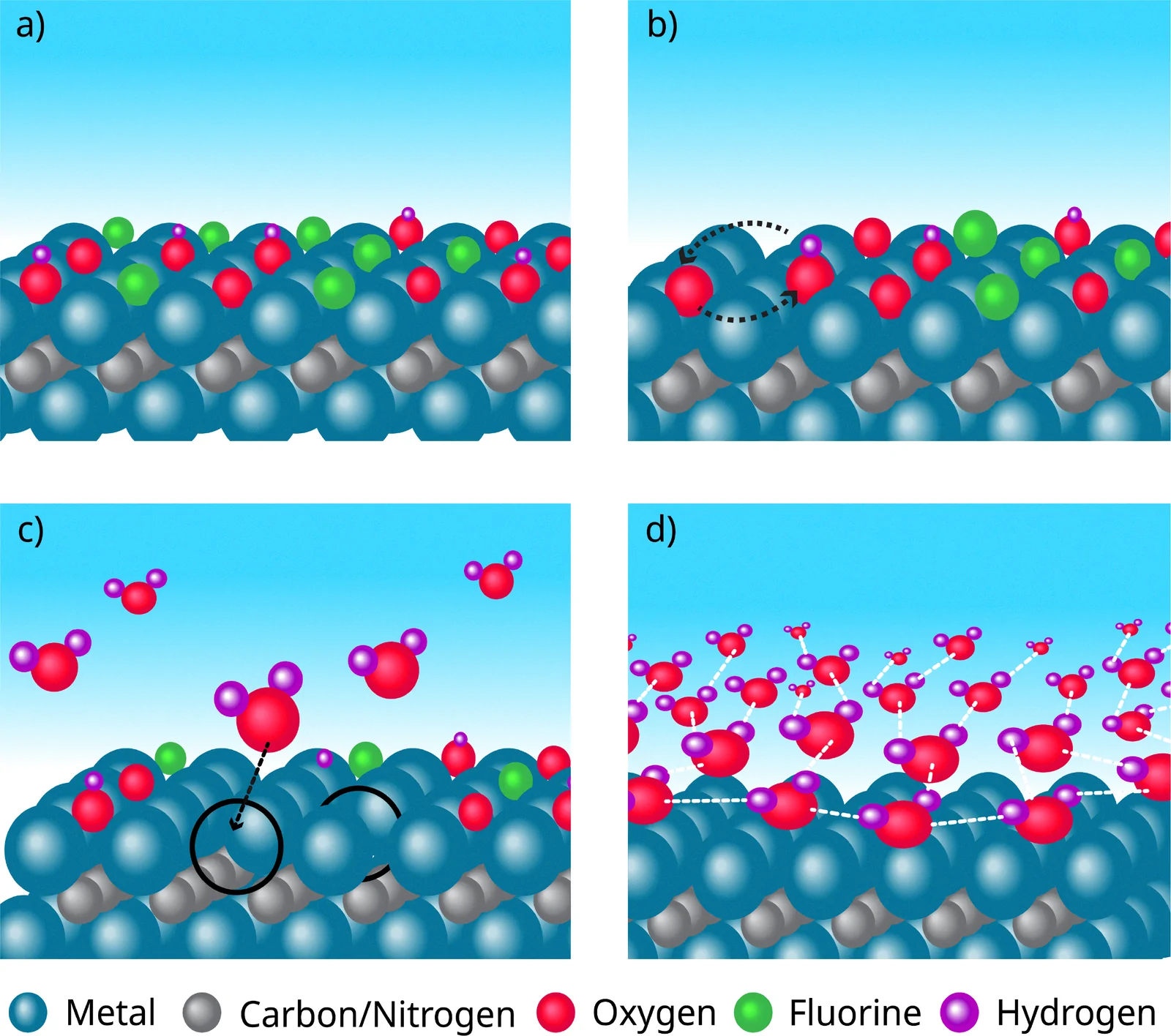
Dynamic nature of the MXene–electrolyte interface under electrochemical conditions. (a) Schematic representation of a MXene surface with heterogeneous distribution of surface terminations (–O, –OH, and –F) in contact with an aqueous electrolyte. (b) Dynamic exchange of surface termination groups at the MXene–electrolyte interface. (c) Local surface reconstruction and defect formation (highlighted by black circles) induced by interactions with water. (d) Structured hydrogen-bond network (dashed lines) of interfacial water near the MXene surface.

Theoretical studies provide a foundation for understanding this behavior. Surface Pourbaix analyses show that the stability of MXene terminations depends strongly on potential and pH, with mixed terminations (*e.g.*, O/OH or O/OH/F) predicted to be stable over HER-relevant electrochemical conditions rather than exclusively idealized single-termination surfaces (Fig. 6a and b).^26,50,51^ These termination distributions can shift with applied bias, directly influencing hydrogen adsorption energetics and reaction pathways. This highlights a key limitation of descriptor models derived from static surface assumptions.

Defects and vacancy sites are increasingly recognized as key contributors to catalytic activity in two-dimensional materials.^52–54^ In MXenes, structural imperfections such as metal or termination vacancies, edge sites, and lattice distortions can modify local electronic structure and adsorption behavior. Theoretical studies suggest that such sites can create unsaturated coordination environments, enhance hydrogen adsorption, and promote charge transfer.^15,55^ Atomistic simulations further indicate that electrochemical conditions can induce localized structural changes. Water adsorption on surface Ti sites has been shown to weaken Ti–C bonds and, in some cases, promote partial extraction of Ti atoms, leading to defect formation and local reconstruction.^55^ The extent of these processes depends on surface chemistry and charge state: O-terminated surfaces enhance water interaction and reactivity, whereas OH- or F-containing and negatively charged surfaces can limit water access through steric and electronic effects.^15,56^ Such sites (Fig. 6c) can act as highly reactive centers where water adsorbs, dissociates, and locally modifies the surface structure. In some cases, water molecules can occupy vacancy sites and stabilize them, while in others they promote further structural transformation depending on the local environment.^57^ Consequently, the catalytic interface is governed not only by average surface composition but also by localized and evolving structural features.

These transformations can have different consequences for catalyst stability. Reversible changes in surface termination or local coordination may generate a favorable catalytic state without permanently disrupting the MXene framework, whereas progressive oxidation, dissolution, or loss of carbide connectivity can alter active-site populations and electronic transport, ultimately leading to irreversible degradation. Importantly, substantial structural transformation may occur without an immediate loss of catalytic activity, making it necessary to correlate electrochemical performance with structural evolution when identifying the catalytically relevant interface.

The electronic structure of MXenes is equally sensitive to electrochemical conditions. Constant-potential simulations show that applied bias drives continuous charge redistribution, leading to shifts in oxidation state, modification of electronic states near the Fermi level, and changes in adsorption energetics.^15^ Protonation–deprotonation processes at the interface further influence electron localization and bonding characteristics, directly affecting the coupling between surface electronic structure and catalytic activity.^14^ As a result, descriptor-relevant quantities such as the density of states near the Fermi level, work function, and hydrogen adsorption energetics can vary with the electrochemical state of the interface rather than remaining fixed during operation.

These effects are further coupled with the behavior of interfacial water. At the MXene–electrolyte interface, hydrogen-bonding networks differ from bulk water and reorganize under applied potential (Fig. 6d).^58^ In particular, cathodic polarization promotes the formation of more strongly hydrogen-bonded water structures, reflecting substantial restructuring of the interfacial solvent layer.^13^ Furthermore, water confined within interlayers, wrinkles, or defect regions of MXenes displays reduced mobility and altered transport behavior compared to bulk water, with intercalated ions further modulating solvent structure and dynamics.^13,59^ These changes in solvent organization directly influence proton transport, interfacial charge distribution, and reaction kinetics, indicating that catalytic processes are governed by a coupled solid–liquid interface rather than by the catalyst surface alone.

Experimental studies provide direct evidence supporting these insights. *In situ* scanning transmission X-ray microscopy (STXM) has revealed water-induced oxidation of surface Ti atoms at the level of individual Ti_3_C_2_T_*x*_ flakes, with significant spatial heterogeneity across the same flake.^13^ This oxidation is partially reversible under acidic conditions, consistent with a contribution from dynamic protonation–deprotonation processes rather than exclusively irreversible structural degradation.^13^ Additional observations show that oxidation behavior and surface chemistry depend strongly on pH and electrolyte composition.^14^ Together, these observations show that the catalytically relevant interface reflects coupled changes in surface termination, local structure, electronic state, and interfacial solvent organization, rather than a single static surface configuration.

### 3.2 Limitations of *ex situ* and static approaches

*Ex situ* characterization techniques are widely used to probe the structure and composition of MXene electrocatalysts; however, they capture surfaces outside the electrochemical environment in which catalysis occurs. Once removed from the electrolyte, the applied potential and interfacial electric field are no longer maintained, leading to desorption or reorganization of weakly bound solvent molecules, adsorbed intermediates, and metastable species. For MXenes, whose surface terminations and bonding environments are highly sensitive to aqueous conditions, this relaxation may result in redistribution of terminations, partial oxidation during drying, or structural changes upon exposure to the ambient atmosphere. As a result, *ex situ* measurements provide valuable compositional insights but do not directly reflect the active state of the catalyst under operating conditions.

This limitation is particularly important when degradation mechanisms are considered. Post-reaction characterization may reveal oxidation, termination redistribution, defect formation, dissolution, or other structural changes, but it cannot determine when these transformations occurred relative to changes in catalytic performance. Consequently, comparison of the initial and final catalyst states alone may not distinguish reversible or activity-promoting reconstruction from irreversible degradation. Establishing this distinction requires the structural evolution of the interface to be correlated temporally with the electrochemical response.

In parallel, theoretical descriptions of MXene electrocatalysis often rely on simplified representations of the interface. Many computational studies employ idealized surfaces with fixed terminations, neutral surface models, and either vacuum or simplified representations of the solvent. While these approaches enable systematic comparisons and facilitate descriptor-based analysis, they may not fully capture key factors such as applied potential, interfacial charge redistribution, and dynamic interactions with the electrolyte. Consequently, descriptor values derived from these models depend strongly on the chosen assumptions and may not accurately represent behavior under realistic electrochemical conditions.

These limitations affect how descriptor–activity relationships are interpreted. Although various descriptors show correlations with HER performance within specific computational or experimental datasets, such relationships may not generalize across different electrolytes, pH values, surface states, or potential ranges. Furthermore, a descriptor–activity correlation does not by itself establish a causal relationship, particularly when the surface state present during operation differs from that assumed in the underlying model or characterization. Without validation under working conditions, it remains difficult to determine which parameters genuinely govern HER activity and which arise from constraints of the characterization or modeling approach.

## 4 Role of *operando* spectroscopy

The relevance of activity descriptors for MXene-based HER catalysis ultimately depends on whether they remain valid at the solid–liquid interface under applied electrochemical bias. Without such validation, descriptor–activity relationships may reflect modeling assumptions rather than the behavior of the catalyst under operating conditions. Descriptors such as Δ*G*_H_, work function, surface termination composition, and defect energetics are typically defined for simplified surface models. However, their interpretation requires direct assessment under conditions where potential, electrolyte, and interfacial interactions collectively define the catalytic state.

Spectroscopic measurements under electrochemical conditions are commonly described as *in situ* or *operando*, depending on how the measurement is performed. In this review, *in situ* refers to spectroscopic measurements carried out under a relevant electrochemical environment or applied bias, without requiring simultaneous measurement of catalytic performance. The term *operando* is reserved for measurements acquired while the electrochemical reaction is actively occurring and the corresponding catalytic response is monitored simultaneously. This distinction is used throughout the following discussion to classify the spectroscopic studies considered in this review.

*Operando* spectroscopic techniques therefore provide a direct means of evaluating descriptor-relevant properties under working conditions. By probing surface chemistry, electronic structure, and local coordination environments at the solid–liquid interface, these methods enable direct observation of the evolving catalytic state. In particular, vibrational spectroscopies such as Raman and infrared spectroscopy probe local bonding environments and interfacial species, while X-ray-based techniques provide complementary information, with XAS probing electronic and local coordination environments and XPS providing chemical-state and surface-composition sensitivity.^60–72^ In the following subsections, representative studies illustrate how these complementary techniques can resolve the evolution of surface terminations, interfacial water structure, and electronic and local structural states of MXenes under electrochemical conditions.

### 4.1 Probing surface termination evolution

A central question in evaluating activity descriptors for MXene-based HER catalysis is how surface terminations evolve under operating conditions and how these changes can be experimentally observed. Because termination identity and distribution directly influence descriptor-relevant surface properties, their evolution must be tracked under electrochemical bias. *Operando* vibrational spectroscopy provides direct access to such transformations by probing changes in the local bonding environment at the MXene surface.

The general principles of *operando* Raman and IR spectroscopy are illustrated schematically in Fig. 7a. In Raman spectroscopy, vibrational modes are probed through the inelastic scattering of light involving virtual states, resulting in energy shifts that reflect changes in the local bonding environment. Because the Raman response depends on the polarizability of the system, it is especially sensitive to variations in surface termination and lattice vibrations. In the following, Raman spectroscopy is used as a representative example to demonstrate how *operando* vibrational techniques probe termination evolution on MXenes.

**Fig. 7:**
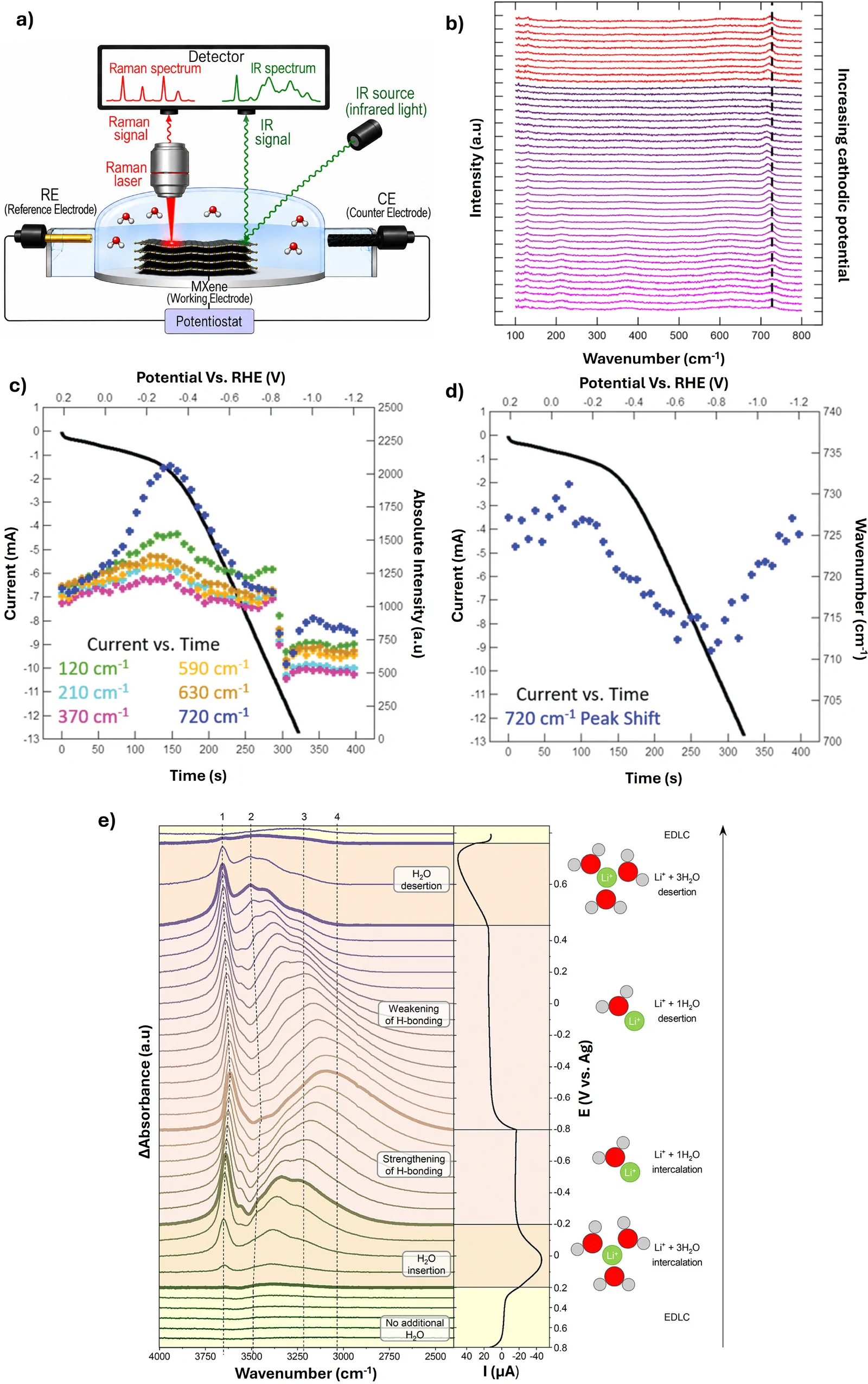
*Operando* vibrational spectroscopic insights into the dynamic evolution of the MXene–electrolyte interface during HER. (a) Conceptual schematic of *operando* Raman and infrared spectroscopy at the solid–liquid interface under electrochemical bias. The illustration highlights the spectroscopic principles and does not represent the actual measurement geometry. (b) *Operando* Raman spectra of Ti_3_C_2_T_*x*_ collected under progressively increasing cathodic potentials during HER in acidic electrolyte, showing the evolution and shift of the characteristic A_1g_ vibrational mode. The dashed line indicates the initial peak position near 735 cm^−1^. (c) Potential-dependent evolution of Raman intensities for characteristic vibrational modes of Ti_3_C_2_T_*x*_ during LSV. (d) Potential-dependent shift of the A_1g_ Raman mode (720 cm^−1^). (e) *Operando* ATR-FTIR spectra of Ti_3_C_2_T_*x*_ showing potential-dependent changes in the O–H stretching region of interfacial water. Components 1–4 correspond to water molecules with increasing hydrogen-bond strength. The electric double-layer capacitance (EDLC) regime and intercalation regions are indicated. Panels (b)–(d) adapted from ref. 73 under the Creative Commons Attribution 3.0 licence (CC BY 3.0). Panel (e) adapted from ref. 58 under the Creative Commons Attribution 4.0 licence (CC BY 4.0).

*Operando* Raman measurements on Ti_3_C_2_T_*x*_ reveal clear potential-dependent changes in the surface environment during HER. Fig. 7b shows *operando* Raman spectra collected during linear sweep voltammetry under progressively increasing cathodic potentials in acidic electrolyte.^73^ The spectra contain several characteristic vibrational modes whose intensities evolve with applied potential during the cathodic sweep (Fig. 7c). The most prominent response is observed for the high-intensity A_1g_ mode near 720–735 cm^−1^. As the potential approaches the HER onset region, the Raman intensity initially increases and reaches a maximum before decreasing again at higher cathodic currents. This behavior has been attributed to changes in electronic polarizability and surface chemistry during HER. The authors attributed the initial enhancement to electron injection into the MXene under cathodic bias, whereas attenuation at higher current densities was associated with H_2_ bubble formation and increased surface coverage by reaction intermediates.^73^

More direct evidence for changes in the termination environment is provided by the potential-dependent shift of the A_1g_ Raman mode, shown in Fig. 7d.^73^ This mode corresponds to out-of-plane vibrations that are highly sensitive to the local surface termination environment. As the applied potential becomes more negative, the A_1g_ peak shifts to lower wavenumbers, indicating a softening of the vibrational mode.^73^ This shift reflects a reduction in the effective restoring force and is consistent with potential-dependent changes in surface termination chemistry. Under HER conditions, electron injection and proton availability can modify the local termination environment, for example through protonation of O-containing terminations. Such changes can alter local bonding and reduce the effective vibrational stiffness, contributing to the observed peak shift. When the potential is reversed, the A_1g_ peak shifts back toward its initial position, indicating that the underlying changes in the local surface environment are at least partially reversible.^73^ The reversible shift therefore supports a dynamically responsive termination environment under electrochemical bias, emphasizing that termination-related activity descriptors should be evaluated under operating conditions.

### 4.2 Probing interfacial water structure and electrolyte effects

In aqueous electrocatalysis, the structure of interfacial water and the arrangement of solvated ions near the catalyst surface play a central role in governing proton transfer, hydrogen adsorption, and reaction kinetics.^74^ Rather than acting as a passive medium, the electrolyte at the solid–liquid interface forms a structured hydrogen-bond network that evolves under applied potential. Understanding how this interfacial solvation environment responds to electrochemical bias is therefore essential for linking catalyst properties to HER activity. *Operando* spectroscopic techniques have increasingly been employed to probe such processes under realistic reaction conditions.^49,75–82^

As described earlier, vibrational spectroscopy is directly sensitive to molecular bonding environments and has been widely used to investigate water structure and adsorbed intermediates at electrochemical interfaces. Infrared (IR) spectroscopy probes vibrational modes through direct absorption between discrete vibrational energy levels and is particularly sensitive to dipole-active species such as hydroxyl groups and interfacial water. In MXenes, the layered architecture further influences the interfacial water environment. Surface terminations interact strongly with water through hydrogen bonding, while interlayer spaces can host solvated ions and water molecules, creating confined electrochemical environments that differ substantially from the bulk solution. Consequently, the hydrogen-bond network and solvation structure near MXene surfaces may influence proton transport pathways and hydrogen adsorption energetics during HER.

Fig. 7e shows *operando* ATR-FTIR spectra of Ti_3_C_2_T_*x*_ recorded during electrochemical cycling in an aqueous LiCl electrolyte, focusing on the O–H stretching region, which is sensitive to the hydrogen-bonding environment of water.^58^ The spectra are decomposed into four characteristic components (labeled 1–4), representing water molecules in different hydrogen-bonding environments within the confined MXene interlayer space. Higher-wavenumber components are associated with weakly hydrogen-bonded or more isolated water molecules, whereas lower-wavenumber components are associated with more strongly hydrogen-bonded water.

As the electrode potential is swept toward negative values, Li^+^ ions intercalate into the MXene interlayer space together with water molecules. This process restructures the hydrogen-bonding environment, as reflected by an increase in the intensity of low-wavenumber components associated with more strongly hydrogen-bonded water.^58^ The spectral evolution therefore indicates an increasing contribution from strongly hydrogen-bonded water under cathodic bias, associated with confinement and ion–water interactions. In contrast, when the potential is reversed toward more positive values, Li^+^ ions and associated water molecules deintercalate, accompanied by a redistribution of spectral intensity toward higher wavenumbers associated with more weakly hydrogen-bonded water species. The potential region without significant faradaic current corresponds to the electric double-layer capacitance (EDLC) regime, where the spectral changes primarily reflect reorientation and redistribution of water molecules and ions rather than faradaic processes.^58^ The reversible evolution of these spectral features shows that interfacial water structure responds dynamically to electrochemical conditions and is closely coupled to ion intercalation processes.

### 4.3 Probing electronic structure and defect-driven structural evolution

*Operando* X-ray absorption spectroscopy (XAS) provides direct access to the electronic and local structural state of transition-metal centers under reaction conditions. In contrast to vibrational techniques, which probe molecular bonding environments, XAS monitors transitions from core electronic levels to unoccupied states (see Fig. 8a). The position and shape of the absorption edge in X-ray absorption near-edge structure (XANES) are sensitive to oxidation state and local chemical environment, while first-derivative spectra can help distinguish changes in overlapping spectral features. At higher energies, extended X-ray absorption fine structure (EXAFS) encodes information on interatomic distances and coordination environments, allowing simultaneous tracking of electronic and structural evolution.

**Fig. 8:**
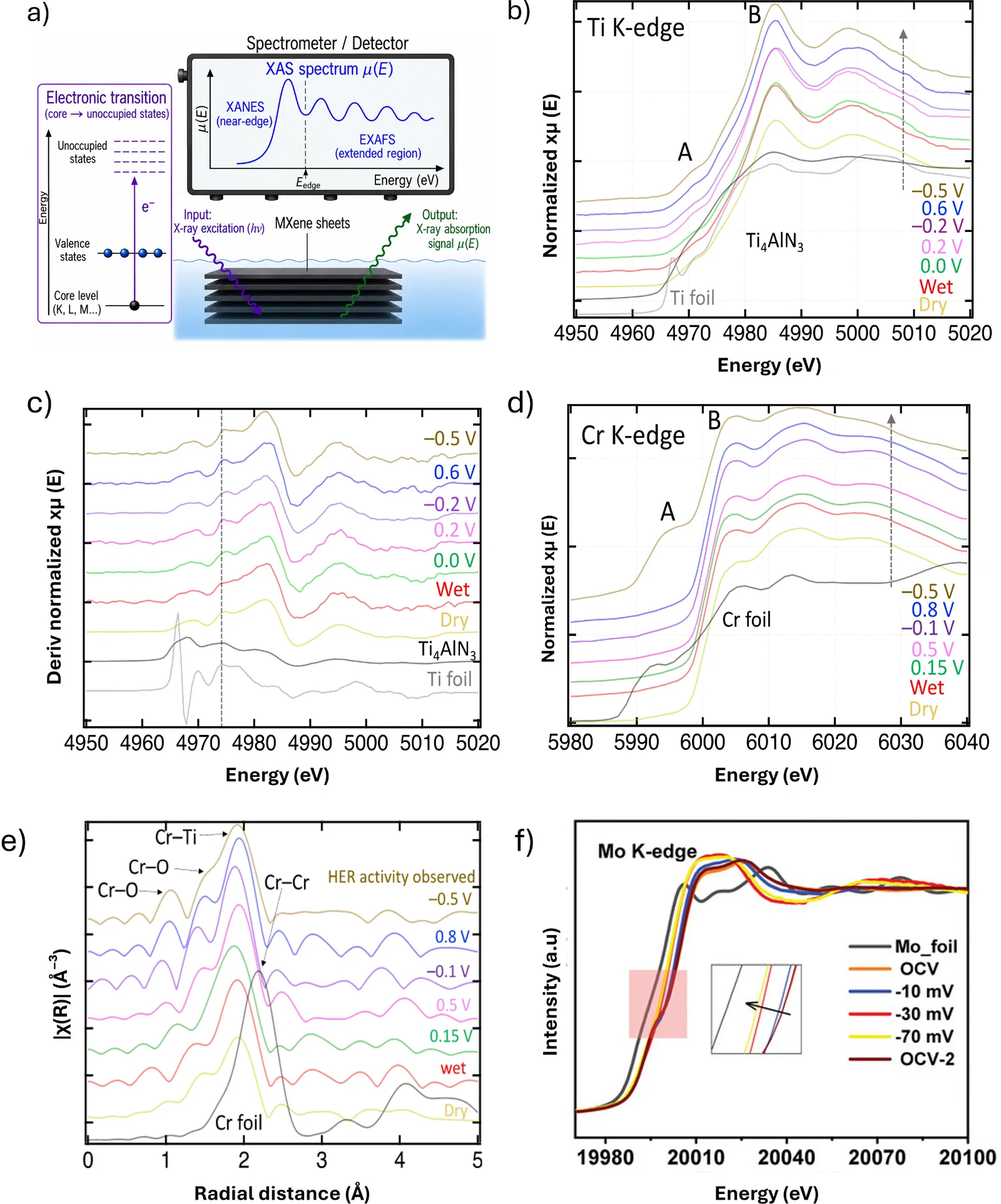
X-ray spectroscopic investigation of electronic and structural evolution in MXene electrocatalysts under HER conditions. (a) Simplified schematic illustration of *operando* XAS, showing electronic transitions from core levels to unoccupied states and the distinction between the XANES and EXAFS regions. (b) Ti K-edge XANES spectra of Ti_4_N_3_T_*x*_ MXene measured under dry, wet, and electrochemically biased conditions. (c) First-derivative Ti K-edge spectra revealing contributions from multiple Ti oxidation states at the MXene surface. (d) Cr K-edge XANES spectra of Cr-alloyed Ti_4_N_3_T_*x*_ MXene, showing potential-dependent spectral evolution during HER. (e) Fourier-transformed Cr K-edge EXAFS spectra showing changes in the local coordination environment and metal–oxygen bonding under electrochemical conditions. (f) Potential-dependent Mo K-edge XANES spectra of Mo cluster-modified Ti_3_C_2_T_*x*_ MXene recorded at different cathodic potentials during HER, indicating potential-dependent electronic restructuring of Mo species at the catalyst–support interface. Panels (b)–(e) are adapted from ref. 83 under the Creative Commons Attribution 4.0 licence (CC BY 4.0). Panel (f) is adapted from ref. 84 with permission from Wiley-VCH, copyright 2024.

*In situ* XANES measurements on Ti-based MXenes reveal the coexistence of different Ti chemical states under electrochemical conditions.^83^ In Fig. 8b, Ti K-edge spectra collected under dry, wet, and electrochemically biased conditions indicate contributions from Ti^2+^, Ti^3+^, and Ti^4+^ species rather than a single oxidation state. These contributions are more clearly distinguished in the first-derivative spectra (Fig. 8c), where multiple features are associated with the different Ti oxidation states.^83^ Importantly, these spectral features show only minor changes with applied potential, indicating that the average electronic state of the Ti centers remains relatively stable over the investigated potential range. The coexistence of multiple Ti oxidation states without pronounced potential-dependent changes further suggests that the presence of mixed oxidation states alone is insufficient to explain the observed HER activity.

To further examine how compositional modification influences electronic evolution under HER conditions, *in situ* XAS was applied to alloyed MXene systems containing transition-metal species such as Mn, Mo, and Cr. For Mn- and Mo-modified MXenes,^83^ XANES spectra show only minor variations in edge position and spectral shape across the applied potential range, indicating that the average oxidation state remains largely unchanged during HER. Despite this apparent electronic stability, Mo-containing systems exhibit measurable catalytic activity, suggesting that subtle changes in local coordination or electronic coupling—rather than pronounced shifts in oxidation state—play a key role in determining catalytic performance.^83^

In contrast, Cr-modified MXenes exhibit clear potential-dependent electronic evolution, as shown in Fig. 8d. Under HER-relevant potentials, the Cr K-edge XANES spectra develop an additional pre-edge feature, which has been attributed to the partial reduction of Cr^3+^ to Cr^2+^.^83^ Notably, this feature appears under HER conditions, linking the observed electronic change to the catalytically relevant electrochemical state. The potential-dependent spectral evolution therefore indicates that the local electronic state of the Cr sites changes under electrochemical bias.^83^

While XANES provides information on oxidation state and electronic configuration, complementary insight into structural evolution is obtained from EXAFS analysis. The Fourier-transformed EXAFS spectra shown in Fig. 8e reveal potential-dependent changes in the local coordination environment of the Cr centers. Three main features are observed, including two Cr–O contributions at approximately 1.0 and 1.5 Å and a Cr–Ti contribution near 2.0 Å. At 0.15 V, close to the Cr redox process, the second Cr–O feature is strongly suppressed, indicating a modification of the oxygen coordination environment around the Cr centers. Under HER conditions (−0.5 V), additional changes in the relative intensities of both the Cr–O and Cr–Ti features are observed compared with the dry and wet reference states. These spectral changes indicate a potential-dependent distortion of the local coordination environment around Cr. The authors attributed this behavior to electrochemically induced structural rearrangements, potentially involving Jahn-Teller distortions that modify Cr–O and Cr–Ti bonding interactions under applied potential.^83^ These observations demonstrate that the local structure of the Cr sites under HER conditions can differ from that of the initial material, highlighting the importance of probing coordination environments under operating conditions.

Beyond structural changes within the MXene lattice, spectroscopic studies under HER conditions have also shown that species supported on MXenes can undergo dynamic reconstruction during HER. A representative example is Mo cluster-modified Ti_3_C_2_T_*x*_, where potential-dependent XAS and Raman spectroscopy were used to monitor the catalyst–support interface under HER conditions.^84^ The potential-dependent Mo K-edge XANES spectra in Fig. 8f show a gradual shift of the absorption edge toward lower energies as increasingly negative potentials are applied, indicating progressive reduction of the Mo species during HER. Complementary EXAFS analysis further revealed a transformation of Mo subnanoclusters toward more isolated atomic configurations under HER conditions.^84^ Importantly, this structural evolution was partially reversed when the applied potential was removed, indicating that the catalyst can reversibly access different structural configurations under electrochemical control. These observations show that the catalytically relevant structure under HER conditions can differ substantially from the as-prepared state as a result of electrochemically driven reconstruction at the catalyst–support interface.

Together, these studies show that the electronic and local structural states of MXene-based catalysts can evolve under electrochemical conditions and that the extent of this response depends strongly on composition and local coordination environment. *In situ* and *operando* XAS therefore provide complementary insight into the catalytically relevant state under applied bias, revealing changes that cannot be captured by static structural models alone.

### 4.4 Challenges and opportunities for *operando* spectroscopy of MXene electrocatalysts

Although *operando* spectroscopic techniques have begun to provide important insights into MXene electrocatalysis, studies that directly probe MXenes under electrochemical operating conditions remain limited. One major challenge arises from the structural and chemical complexity of MXenes themselves. Practical MXene systems frequently contain mixed surface terminations, intercalated ions, defects, partially oxidized regions, and spatially heterogeneous surface chemistries that can change under applied bias. This complexity complicates spectral interpretation because several interfacial processes may occur simultaneously during HER. As a result, separating changes directly associated with the catalytic state from concurrent electrolyte-induced or structural transformations remains challenging.

Another important limitation is that individual *operando* techniques provide only partial information about the catalyst–electrolyte interface. Vibrational spectroscopies such as Raman and IR spectroscopy are highly sensitive to molecular bonding environments and interfacial species but provide limited direct information on the electronic states of transition-metal centers. In contrast, XAS provides complementary information on oxidation state and local coordination environment, but typically requires synchrotron radiation and specialized electrochemical cells. Consequently, no single *operando* technique can fully resolve the coupled evolution of surface terminations, interfacial solvation, electronic structure, and local structure during electrocatalysis.

A more complete description of the working MXene–electrolyte interface therefore requires the combination of *operando* spectroscopies that probe different aspects of the same evolving system. As summarized in Table 2, vibrational techniques primarily provide information on local bonding and the molecular environment, whereas X-ray-based spectroscopies probe metal-centered electronic structure, local coordination, and surface chemical states. Combining these complementary capabilities can relate changes in interfacial water and surface chemistry to the accompanying electronic and structural evolution of the catalyst.^85^

**Table 2:** Complementary *operando* spectroscopic techniques for probing the MXene–electrolyte interface during HER

Technique	Information probed	Relevance to MXene HER	Strengths	*Operando* limitations	Complementary role
Raman^73^	Vibrational modes, lattice vibrations, and local bonding environment	Tracks termination-sensitive vibrations and potential-dependent structural changes	Sensitive to surface chemistry and lattice evolution	Spectral changes can have multiple origins; gas evolution may interfere with signal collection	Links surface and lattice changes with electronic and chemical-state information from XAS and XPS
IR^58^	Molecular vibrations, functional groups, and hydrogen bonding	Probes interfacial water, hydrogen-bond restructuring, and molecular species near the MXene surface	Sensitive to interfacial water and molecular bonding	Water and electrolyte absorption can complicate spectral interpretation; limited sensitivity to metal electronic states	Connects the molecular environment with surface and electronic changes observed by Raman, XAS, and XPS
XANES^83,84^	Electronic structure, oxidation-state changes, and local symmetry	Tracks potential-dependent changes in transition-metal electronic states	Element-specific and sensitive to metal-centered electronic changes	Requires synchrotron radiation and specialized *operando* cells; contributions from heterogeneous states may overlap	Links electronic-state changes with local structure from EXAFS and surface chemistry from XPS and Raman.
EXAFS^83,84^	Local coordination, neighboring atoms, and interatomic distances	Tracks changes in local coordination and structural reconstruction	Element-specific and sensitive to local structure without requiring long-range order	Structural interpretation is model-dependent; heterogeneous environments can complicate fitting	Provides structural context for electronic changes observed by XANES and surface changes detected by Raman and XPS
XPS^69,75,86–89^	Elemental composition, chemical states, and binding-energy shifts	Probes surface terminations, oxidation states, interfacial chemical changes and interfacial electrostatic potentials	High chemical specificity and surface sensitivity	Electrolyte attenuates photoelectrons; *operando* measurements require specialized cell geometries	Links surface chemical states with electronic and structural changes observed by XAS and vibrational spectroscopy

Recent developments in near-ambient-pressure and hard X-ray photoelectron spectroscopy provide promising opportunities for probing buried solid–liquid interfaces under electrochemical conditions.^69,75,86–89^*Operando* XPS has the potential to monitor surface terminations, chemical states, adsorbed intermediates, and electrolyte-induced changes in surface chemistry with high chemical specificity. Extending these approaches to MXene electrocatalysts could provide valuable insight into the evolution of the active interface during HER, particularly when combined with complementary *operando* Raman, IR, or XAS measurements. Improved temporal resolution may further help distinguish transient interfacial changes from persistent structural transformations and correlate their evolution with the electrochemical response.

In addition to the interpretative challenges discussed above, *operando* investigation of MXenes presents several practical difficulties related to the measurement environment and electrode configuration. Electrolyte layers can attenuate incident or detected signals, while gas evolution during HER can destabilize the electrochemical interface and interfere with signal collection. MXenes are also commonly investigated as thin films, composite electrodes, or catalyst inks containing binders, conductive additives, and residual synthesis species, which may contribute additional spectroscopic signals or alter the interfacial chemistry. Careful electrode preparation and electrochemical cell design are therefore essential for obtaining reliable *operando* measurements.

Overall, the key challenge is not simply to observe changes in MXenes under electrochemical conditions, but to determine which of these changes define the catalytically relevant interface. This requires resolving chemically heterogeneous surfaces, maintaining a representative solid–liquid interface during measurement, and integrating complementary probes without compromising electrochemical relevance. Equally important is distinguishing reversible formation of an active state from irreversible degradation and correlating these processes directly with HER performance. Addressing these challenges will be essential for validating proposed activity descriptors against the working catalyst and establishing reliable structure–activity–stability relationships for MXene electrocatalysts.

## 5 Conclusions and outlook

MXenes have attracted significant attention as electrocatalysts for the HER because of their tunable electronic structure, diverse surface chemistry, and high electrical conductivity. Over the past decade, numerous theoretical studies have proposed activity descriptors to rationalize HER performance on MXene surfaces. These include hydrogen adsorption energetics, surface termination chemistry, the electronic structure of transition-metal centers, and defects or vacancies that can modify local adsorption and electronic properties. Together, these descriptors provide useful frameworks for interpreting catalytic trends and guiding the design of MXene-based electrocatalysts.

At the same time, the catalytic interface of MXenes under electrochemical conditions is inherently dynamic. During HER, interactions between the catalyst surface, electrolyte species, applied potential, and reaction intermediates can modify surface terminations, alter the electronic structure of metal atoms, reorganize interfacial water networks, and generate new defect sites. Such transformations may influence both catalytic activity and stability, emphasizing that electrochemical performance cannot always be interpreted from the initial or post-reaction material state alone.

*Operando* spectroscopic techniques provide a crucial bridge between theoretical models and experimental observations. Recent studies have begun to reveal potential-dependent changes in surface chemistry, electronic structure, interfacial water organization, and local structure. Such observations are essential for evaluating whether descriptors derived from static models remain representative of the working catalytic interface and for establishing reliable structure–activity relationships.

Despite this progress, *operando* investigations of MXene electrocatalysts remain relatively limited compared with other catalytic material systems. Significant challenges remain in probing buried solid–liquid interfaces, disentangling coupled interfacial processes, and correlating spectroscopic signatures directly with catalytic activity. Future progress will require improved electrochemical cell architectures, complementary *operando* techniques, and closer integration between spectroscopy, electrochemistry, and theoretical modeling.

Looking forward, integrating *operando* spectroscopy with descriptor-based catalyst design offers a promising pathway toward a more realistic understanding of MXene electrocatalysis and the rational development of improved HER catalysts.

### 5.1 Take-home messages

• HER activity in MXenes reflects coupled structural, chemical, and electronic factors rather than a single universal descriptor.

• The MXene–electrolyte interface evolves under electrochemical conditions, meaning that descriptors derived from static structures may not fully represent the working catalytic state.

• Catalytic activity and electrochemical stability do not necessarily imply preservation of the original MXene structure; reversible reconstruction should therefore be distinguished from irreversible degradation when interpreting catalyst performance.

• *Operando* spectroscopy provides a direct route for connecting descriptor-based models with the evolving catalytic interface and establishing more reliable structure–activity–stability relationships under HER conditions.

## Conflicts of interest

There are no conflicts to declare.

## Data Availability

No primary research results, software or code have been included and no new data were generated or analysed as part of this review.
